# DAXX in Metabolic, Aging, and Immune Regulation: Recent Insights

**DOI:** 10.3390/cells15050425

**Published:** 2026-02-27

**Authors:** Jie Zhou, Liyan Zhao, Qinhui Tuo

**Affiliations:** 1 Key Laboratory of Vascular Biology and Translational Medicine, Medical School, Hunan University of Chinese Medicine, Changsha 410208, China; 20243847@stu.hnucm.edu.cn; 2 School of Pharmacy, Hunan University of Medicine, Huaihua 418000, China; 3 Key Laboratory for Quality Evaluation of Bulk Herbs of Hunan Province, School of Pharmacy, Hunan University of Chinese Medicine, Changsha 410208, China; zhaoliyan@nxmu.edu.cn

**Keywords:** DAXX, metabolism, aging, immunity, inflammation

## Abstract

Death domain-associated protein 6 (DAXX) was originally identified as a key regulator of Fas receptor-mediated apoptosis. Recent studies have found that it plays a central role in many biological processes, such as cell metabolism, aging and immunity. DAXX, through its nuclear localization and epigenetic regulatory capabilities, participates in the maintenance of metabolic homeostasis, DNA damage repair, and telomere stability, and modulates immune responses by regulating the transcriptional programs of immune-related genes. This review systematically summarizes recent studies that reveal in various biological processes, including cell metabolism, aging, and immunity, and explores its potential as a therapeutic target, providing a theoretical basis for the study of related diseases and clinical interventions.

## 1. Introduction

Metabolic homeostasis, cellular aging, and immune regulation are vital biological processes essential for overall health. Their dynamic equilibrium plays a crucial role in maintaining tissue function and the stability of the internal environment. Disruptions in these processes are closely associated with the development of various chronic diseases [1,2]. These processes are not isolated; instead, they are interconnected through nuclear mechanisms such as chromatin remodeling and transcriptional regulation, collectively shaping the cellular response to metabolic fluctuations and stress stimuli. Recent research indicates that DAXX, a multifunctional nuclear regulatory protein, plays a central role in these processes through its chromatin remodeling and epigenetic regulatory functions [3,4]. DAXX is involved not only in regulating energy metabolism [5], but also in the DNA damage response, telomere maintenance [6], and the preservation of immune homeostasis [7]. This review focuses on the nuclear localization of DAXX and systematically examines its molecular mechanisms and roles in metabolism, aging, and immune regulation, aiming to clarify its pivotal role in both physiological and pathological conditions.

## 2. Basic Functions of DAXX

### 2.1. Molecular Structure and Functional Domain of DAXX

The DAXX protein is encoded by the DAXX gene, which is located on the short arm of human chromosome 6 (6p 21.3). The gene spans approximately 80 kb and consists of 16 exons. Its protein product is composed of 740 amino acids, with a molecular weight of approximately 81 kDa, and is highly conserved across various mammalian species [8]. The primary sequence of DAXX contains both acidic and basic regions, which facilitate interactions with a variety of proteins [9]. Structurally, DAXX lacks a typical repetitive motif; its secondary structure is predominantly composed of helices and disordered regions, with the C-terminal being rich in intrinsic disorder (IDR), while the N-terminal forms a stable folded domain [10,11].

**Figure 1:**
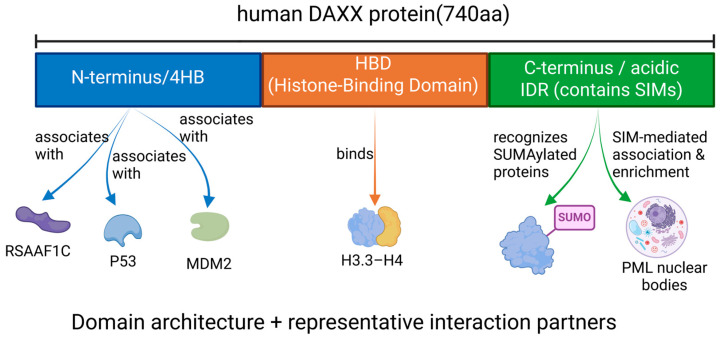
Domain architecture and representative interaction partners of human DAXX protein. This schematic illustrates the key structural domains of DAXX, including its interactions with various binding partners (such as P53, MDM2, and histones), as well as its SUMO-mediated interaction with PML nuclear bodies. Colors do not imply activation or inhibition.

At the functional domain level, the N-terminus of DAXX harbors a conserved four-helix bundle (4HB) that binds to signaling molecules, including Ras association domain family member 1C (RASSF1C), p53, and mouse double minute 2 homolog (MDM2), thereby regulating apoptosis and transcriptional repression [12]. Its central region, the histone binding domain (HBD), consists of six α helices and is the core structure that binds the H3.3–H4 dimer and performs histone chaperone functions [13]. The C-terminal acidic region is involved in chromatin remodeling and gene silencing while enhancing H3.3/H4 affinity [12,13]. In addition, two small ubiquitin-modifying (SUMO) interaction motifs (SIMs) enable DAXX to bind to SUMOylated proteins, thereby regulating subcellular localization and transcriptional regulatory activity [10,14] (Figure 1).

In terms of subunit interactions, DAXX primarily forms complexes with H3.3/H4 dimers, which dictates its specific localization in heterochromatin, telomeres, and centromeres, contributing to the higher-order structure of chromatin and the maintenance of genome stability [13]. There is a synergistic effect between the different domains of DAXX; for instance, the histone-binding domain (HBD) works in concert with the C-terminal acidic region to enhance the affinity for H3.3/H4 [12]. Additionally, SIM-mediated SUMO interactions facilitate the enrichment of DAXX in PML nuclear bodies, where it plays a role in chromatin silencing and DNA repair [11,15].

### 2.2. Nuclear Localization and Function of DAXX

The diverse functions of DAXX are highly dependent on its nuclear localization. While it primarily mediates apoptotic signaling in the cytoplasm, in the nucleus, DAXX plays critical roles by binding to chromatin, participating in transcriptional regulation, and maintaining genomic stability. This makes it a classic example of a ‘nuclear localization-dependent’ regulatory factor [16,17,18].

At the chromatin level, DAXX is a key partner in the deposition of the histone variant H3.3. It works with associated complexes to specifically load H3.3 into telomeres, centromeres, and repetitive DNA regions, thereby maintaining heterochromatin stability and long-term genomic integrity [16,17,18]. Impaired nuclear localization disrupts the deposition pattern of H3.3, compromises telomere function, and leads to abnormal accumulation of DNA damage signals, ultimately destabilizing genome homeostasis [6,19,20]. Recent studies have shown that ATR (Ataxia telangiectasia and Rad3-related protein)-dependent signaling is crucial for DAXX’s localization to the centromere, a mechanism that helps maintain chromatin markers and regional identity [21]. Additionally, DAXX facilitates the establishment of specific epigenetic modifications, such as the deposition of H3K9me3, providing a new mechanism for gene silencing and suppression of repetitive sequences [19]. These findings suggest that DAXX’s nuclear localization not only ensures the maintenance of chromatin structure but also plays a unique role in regulating epigenetic modifications.

The nuclear localization of DAXX is also evident in its aggregation within the nucleus. Previous studies have shown that DAXX is highly enriched in nuclear substructures, such as PML nuclear bodies, where it regulates apoptotic signaling and transcriptional repression [22,23]. Further studies revealed that the SUMO-interacting motif (SIM) is crucial for maintaining its nuclear localization and stability [14,24,25]. The absence or disruption of SIM impairs DAXX’s ability to effectively accumulate in PML nuclear bodies, causing it to diffuse throughout the nucleus and weakening its associated functions. Recent studies have also shown that DAXX undergoes nuclear redistribution during viral latency and reactivation, and exhibits reversible aggregation driven by liquid–liquid phase separation, which reflects the close relationship between its nuclear localization and stress response [26]. At the same time, the level of SUMO modification directly affects the nuclear body residence and stability of DAXX, and excessive or insufficient modification will destroy its localization and function [15,27].

In addition to its role in intranuclear structures such as chromatin and PML nuclear bodies, the nuclear localization of DAXX determines its functional performance in transcriptional regulation. For example, it can bind to and inhibit the activity of some transcription factors in the nucleus, thus regulating the expression of downstream genes [28]. These studies reveal that the transcriptional regulation of DAXX depends on nuclear localization rather than simple protein interactions. Recent studies have also highlighted the relationship between nuclear localization and its dynamic plasticity: DAXX can rapidly adjust its distribution under different environmental and signaling conditions, switching from a static chromatin partner to a dynamic transcriptional regulator [24,26]. This feature provides an important basis for explaining its diversified functions.

Nuclear localization abnormalities are often associated with pathological processes [29,30]. As a result of mutations found in some tumors that disrupt key domains necessary for DAXX to maintain its nuclear localization, DAXX is unable to form normal aggregated structures in the nucleus, and its functions related to chromatin loading are impaired. This impairment in function further triggers a block in the chromatin deposition process and perturbs downstream transcriptional regulation [19]. In addition, recent studies on nuclear body structure have shown that the aggregation and distribution of DAXX are regulated by a variety of signals, and its abnormalities may become the starting point of chromatin opening or genomic instability [31,32,33].

These results collectively suggest that nuclear localization is not merely a phenomenon of DAXX localization, but also a key factor determining whether its function can be normally exerted.

## 3. Regulatory Mechanism of DAXX in Metabolism

Energy metabolism is a fundamental process essential for maintaining life activities and tissue homeostasis, involving the acquisition, utilization, and storage of carbohydrates, lipids, and proteins. Imbalances in this process can impair cellular function, disrupt tissue homeostasis, and affect overall health, contributing to the development of various chronic diseases [34,35]. Recent studies have increasingly recognized that DAXX has emerged as a regulator of metabolic gene programs through its nuclear chromatin functions, thereby influencing glucose and lipid metabolism [15,32,36].

### 3.1. Lipid Metabolism

Lipid metabolism is a central aspect of energy metabolism and signal transduction, encompassing processes such as cholesterol metabolism, fatty acid synthesis, oxidation, and triglyceride synthesis [37]. These metabolic pathways are highly coordinated under physiological conditions, working in concert to maintain cellular homeostasis [38]. However, disruptions in any step of these pathways can lead to lipid accumulation and metabolic dysfunction, contributing to conditions such as obesity, diabetes, non-alcoholic fatty liver disease (MASLD/MASH), and cancer [39,40]. During this process, DAXX integrates epigenetic regulation with the transcriptional activity of metabolic genes through its nuclear localization and chromatin-regulatory functions. DAXX modulates the activation and inhibition of lipid metabolism-related genes by interacting with PML nuclear bodies, ATRX, and various transcriptional complexes, thereby regulating lipid homeostasis in response to changes in the metabolic environment [12,21].

Studies show that DAXX is crucial for cholesterol and fatty acid synthesis. These processes are initiated when sterol regulatory element-binding protein (SREBP) precursors are cleaved and enter the nucleus [5,41]. DAXX, through its nuclear localization, interacts with SREBP1/2 to regulate the expression of metabolic genes. Its activation state directly influences the transcription levels of synthesis-related genes and directs metabolic processes [5]. In the cholesterol synthesis pathway, SREBP exists as a precursor in the endoplasmic reticulum. When cellular cholesterol levels drop, the chaperone protein SREBP cleavage-activating protein (SCAP) transports SREBP to the Golgi apparatus, where it is cleaved by Site 1 protease (S1p) and Site 2 protease (S2p). The N-terminal fragment is then released into the nucleus, driving the transcription of key genes such as HMGCR, HMGCS1, and LDLR [42,43,44], thereby promoting cholesterol production. Its nuclear transcriptional activity is regulated by chromatin accessibility and the nuclear microenvironment, such as the PML nuclear bodies [15,33]. During this process, DAXX uses its SUMO-binding domain to interact with SREBP2, stabilizing its binding to the promoters of lipid metabolism-related genes. This significantly upregulates the expression of HMGCR and HMGCS1, promoting cholesterol accumulation [5]. This mechanism was primarily demonstrated in triple-negative breast cancer cells such as MDA-MB-231 [5]. In addition, SREBP1 also plays a key role in fatty acid synthesis. The activated fragment formed after SREBP1 cleavage can up-regulate ACC, FASN, SCD1, ACLY and other genes, promote the supply of acetyl-Coa to malonyl-Coa, and the synthesis and desaturation of long-chain fatty acid chains [45]. DAXX enhances the transcriptional activity of SREBP1/2 by virtue of its SIM2 domain binding to SUMO, thereby promoting fatty acid synthesis [5,46]. Consistently, disruption of the DAXX SIM2 domain blocks this pathway, resulting in reduced lipogenesis and suppressed cell proliferation [5]. At the same time, the effect of DAXX is also closely related to androgen receptor (AR). In androgen-dependent prostate cancer cells, testosterone or dihydrotestosterone (DHT) binds to cytoplasmic androgen receptor (AR), inducing conformational changes, dissociation from chaperone proteins, and subsequent nuclear translocation of the full-length receptor. Once in the nucleus, activated AR binds androgen response elements (AREs) and promotes the transcription of lipid metabolic regulators, including SREBF2, leading to increased expression of HMGCR, HMGCS1, ACC and FASN in prostate cancer cells. This AR-driven transcriptional program increases cholesterol and fatty acid synthesis, thereby supporting membrane biosynthesis and tumor cell proliferation in the prostate cancer context [47,48]. Notably, DAXX can antagonize this pathway by interacting with the N-terminal and DNA-binding domains of AR, thereby attenuating AR-mediated activation of SREBP2. In LNCaP prostate cancer cells, SUMO modification of AR further enhances its association with DAXX, strengthening their cooperative repression at chromatin platforms and ultimately suppressing SREBP2 activation and downstream metabolic gene expression [28,49,50].

**Figure 2:**
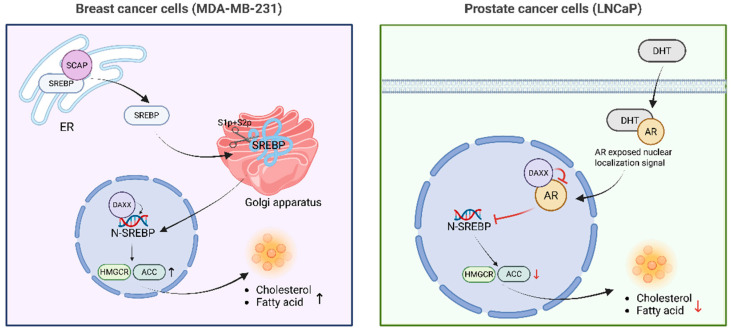
Bidirectional regulation of lipid metabolic programs by DAXX in distinct tumor contexts. In breast cancer cells (e.g., MDA-MB-231), DAXX interacts with SREBP1/2 and promotes transcription of lipogenic enzymes such as HMGCR and ACC, thereby enhancing cholesterol and fatty acid synthesis to support tumor growth. In contrast, in prostate cancer cells (e.g., LNCaP), DAXX antagonizes androgen receptor (AR)-mediated activation of SREBP2, suppressing downstream lipogenic gene expression and reducing lipid synthesis. These findings illustrate that DAXX functions as a context-dependent regulator of metabolic gene expression, coordinating anabolic lipid programs according to tumor-specific signaling environments. Black arrows indicate the direction of the proposed pathway/interaction. Red T-bars denote inhibitory effects. ↑ and ↓ indicate increased and decreased levels, respectively. Colored panels distinguish the two tumor contexts shown and are used for visualization only.

This bidirectional regulation may be due to the different epigenetic status of DAXX in different cellular environments. The level of SUMOylation and its spatial distribution in the nuclear body of PML may determine the accessibility and interaction stability of DAXX to different transcriptional programs (such as SREBP or AR-related pathways) [15,51]. In addition, the position of DAXX in the metabolic signaling pathway may be dynamically regulated by energy sensing mechanisms such as AMP-activated protein kinase (AMPK) and mechanistic target of rapamycin (mTOR), so that it tends to promote synthesis under high metabolic pressure and to inhibit feedback under steady-state conditions [52]. Thus, rather than functioning as a simple activator or inhibitor of lipid metabolism, DAXX appears to act as a context-dependent metabolic integrator, coordinating anabolic or catabolic programs according to cellular demands (Figure 2).

The DAXX–SREBP1/2 axis plays a crucial role in regulating the upstream synthesis of cholesterol and fatty acid metabolism, and its influence may extend to lipid storage [53]. The synthesis of triacylglycerol (TAG) is dependent on diacylglycerol acyltransferase 1/2 (DGAT1/2), and the transcription of DGAT1/2 is indirectly regulated by SREBP1 [45,54,55]. DAXX enhances the transcriptional activity of SREBP1 [5], suggesting it may indirectly regulate DGAT1/2 expression, thereby affecting TAG production and lipid droplet accumulation. Conversely, when the DAXX–SREBP axis is disrupted, the supply of fatty acids and cholesterol is reduced, leading to diminished TAG production. This indirect regulation through substrate accumulation and coordinated metabolic pathway transmission suggests that the DAXX–SREBP mechanism plays a role in lipid storage and long-term metabolic adaptation, though its specific function requires further experimental validation.

Cholesterol homeostasis is also regulated by efflux and transport pathways [56]. DAXX has been shown to upregulate caveolin-1 expression, enhancing cholesterol transport to high-density lipoproteins (HDLs) or plasma membranes. This promotes cholesterol efflux and prevents lipid deposition [57,58,59]. In summary, DAXX promotes cholesterol efflux by upregulating caveolin-1 and coordinates cholesterol transport through nuclear localization-dependent transcriptional control.

Fatty acid oxidation is a key metabolic process for maintaining energy homeostasis, primarily regulated by AMPK and its downstream target, carnitine palmitoyltransferase 1A [60]. During energy deprivation, AMPK is activated, inhibiting lipid synthesis and promoting fatty acid oxidation in mitochondria, thereby preventing lipid accumulation [61]. In a high-fat diet-induced obesity mouse model, DAXX overexpression activates the AMPK-related kinase MPK38, with its N-terminal (1–440 aa) binding to the C-terminal (270–643 aa) of MPK38 to enhance its stability and activation. Activated MPK38 exerts AMPK-like effects through the ASK1/TGF-β/p53 signaling pathway, significantly increasing fatty acid oxidation levels [62]. This mechanism reduces lipid droplet accumulation in hepatocytes and lowers serum triglyceride and total cholesterol levels [62], thereby playing a protective role in maintaining overall metabolic homeostasis. These findings offer new insights into DAXX’s role in metabolic diseases such as obesity and fatty liver.

### 3.2. Glycometabolism

Imbalances in glucose metabolism are a key pathological factor in various diseases, including insulin resistance, obesity, type II diabetes, cardiovascular disease, and cancer-related metabolic reprogramming [54]. Glucose uptake, glycolysis, and gluconeogenesis work in a coordinated manner to maintain systemic glucose homeostasis and energy supply, and they contribute to disease progression through interactions with transcription factors and signaling pathways [63,64]. At these glycometabolic nodes, DAXX regulates the homeostasis of metabolic pathways by controlling the chromatin accessibility of key glycometabolic enzymes and transcription factors. This epigenetic regulation allows DAXX to play a crucial role in the transcriptional regulation of the glycometabolic network, contributing significantly to metabolic adaptation and the progression of metabolic diseases.

Glucose transport into cells is primarily mediated by glucose transporters (GLUTs), with GLUT4 being the most insulin-sensitive transporter in skeletal muscle and adipose tissue [65,66]. The intracellular recycling of GLUT4 and its membrane translocation depend on a microtubule network regulated by kinesin 5B and JNK1 [67]. Notably, DAXX can directly interact with GLUT4, and both proteins can be modified by SUMOylation. In the insulin-stimulated transport system, DAXX acts as a scaffold protein, cooperating with JNK1 and KIF5B to form a complex. This complex participates in the transport of GLUT4 vesicles and enhances the membrane translocation efficiency of GLUT4. Thereby further regulating glucose intake. Loss of DAXX leads to the retention of GLUT4 in endosomal compartments, thereby impairing transmembrane glucose transport and ultimately disrupting energy acquisition and utilization in tissues [68,69,70].

Glycolysis is a major pathway for rapid energy production in cells, with key rate-limiting steps controlled by hexokinase (HK), phosphofructokinase 1 (PFK1), and pyruvate kinase (PK) [71] HK2 and PKM2 are particularly important in tumor metabolic reprogramming [72]. Studies have shown that in small cell lung cancer, DAXX binds to long non-coding RNA CALML3-AS1, enhances GLUT4-mediated glucose uptake, upregulates HK2 and PKM2 levels, accelerates glycolysis and lactate production, provides sufficient ATP and metabolic intermediates for tumor cells, and supports their rapid proliferation and survival [68]. The role of DAXX in the regulation of glucose metabolism depends not only on its interaction with metabolism-related molecules and complexes, but also on its nuclear localization and regulatory function at the chromatin level. In the mother-infant model of type I diabetes mellitus, the abnormal expression of DAXX may be significantly related to H3K4me3 modification in the promoter region of glycolytic genes [73]. H3K4me3 is a typical transcriptional activation marker, which occurs in the promoter region of the nucleus, and its state directly affects the expression of metabolism-related genes [74]. H3K4me3 has been reported to undergo alterations in hyperglycemic conditions, which are accompanied by remodeling of chromatin accessibility and transcriptional activity programs in promoter regions, thus driving changes in metabolism-related gene expression profiles [75,76]. Given that DAXX possesses histone chaperone and chromatin regulatory functions [6], whether it influences the transcriptional activity of glucose metabolism genes by participating in or regulating H3K4me3-related chromatin states remains to be further systematically studied.

Gluconeogenesis occurs predominantly in the liver, where the rate of conversion of substrates such as lactate, glycerol, and amino acids into glucose is tightly regulated by phosphoenolpyruvate carboxykinase (PEPCK) and glucose-6-phosphatase (G6Pase) [77]. In mice with hepatic DAXX deficiency, hepatic PEPCK and G6Pase expression was increased, consistent with enhanced gluconeogenesis. Adenoviral restoration of DAXX suppressed PEPCK and G6Pase expression and improved glycemic control [62].

It can be seen that DAXX has many regulatory functions in glucose metabolism. It interacts with GLUT4 and related complexes to affect glucose uptake and regulate the expression of key enzymes in glycolysis, thus changing the activity of downstream metabolic pathways. In the liver, DAXX inhibits gluconeogenesis by negatively regulating PEPCK and G6Pase. The association of DAXX with H3K4me3 modification in the promoter region of glycolytic genes suggests that its role is not only dependent on complex assembly and signal regulation at the cytoplasmic level, but also closely related to nuclear localization and epigenetic regulation. Therefore, DAXX emerges as a key regulator of glucose homeostasis and metabolic reprogramming in the contexts described, and provides a mechanistic entry point for research into glucose metabolism–related diseases.

### 3.3. Antioxidant Response

Antioxidant response is an important defense process for cells to cope with metabolic disorders and environmental stresses, and plays a key role in many diseases [78]. It is usually induced by factors such as nutrient loading, hypoxia or excessive reactive oxygen species (ROS), thus breaking the metabolic homeostasis [79,80]. Under this condition, cells initiate defense mechanisms, such as removing damaged cells, activating antioxidant defense and energy redistribution, to maintain survival and function [81]. It was found that the expression of DAXX was often induced in this process, accompanied by translocation from nucleus to cytoplasm, which provided a spatial basis for its regulation of multiple signaling pathways [82].

**Figure 3:**
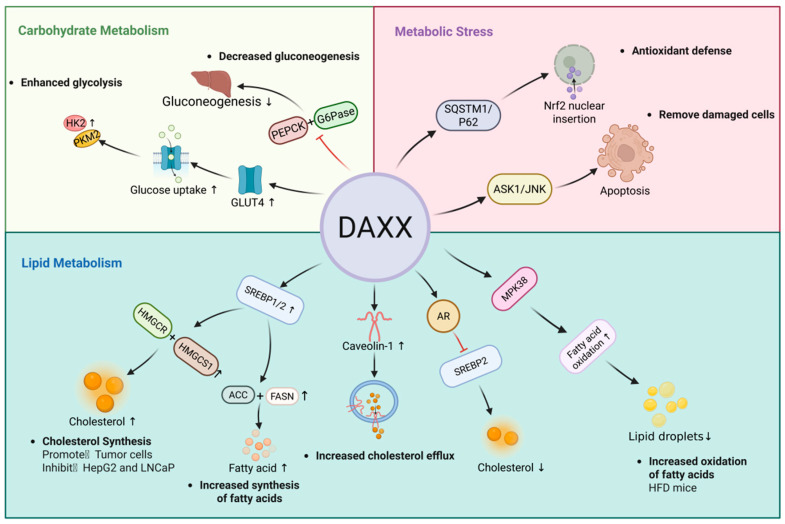
DAXX regulates metabolic pathways through nuclear and cytoplasmic mechanisms. In the nucleus, DAXX cooperates with SREBP1/2 to enhance transcription of cholesterol and fatty acid synthesis enzymes, including HMGCR and HMGCS1. DAXX also modulates lipid metabolism in an androgen receptor-dependent context. In the cytoplasm, DAXX activates the AMPK-related kinase MPK38, promoting fatty acid oxidation. DAXX further participates in glucose metabolism by facilitating GLUT4 trafficking via the JNK1–KIF5B complex and suppressing hepatic gluconeogenesis through regulation of PEPCK and G6Pase. Under metabolic stress, cytoplasmic DAXX promotes p62-mediated Nrf2 activation by facilitating KEAP1 sequestration. Black arrows indicate the direction of the proposed pathway/relationship. Red T-bars denote inhibitory effects. ↑ and ↓ indicate increased and decreased levels, respectively. Colored panels are used to group functional modules for clarity and have no quantitative meaning.

On the one hand, ROS are able to activate the ASK1–JNK signaling axis, thereby inducing apoptosis to clear damaged cells [83]. In the cytosolic environment, DAXX can act as a binding scaffold for ASK1, promote the activation of JNK pathway, promote apoptosis, and help the body to remove severely damaged cells in time and maintain tissue homeostasis [9]. On the other hand, cells also enhance stress tolerance through antioxidant pathways [84]. The selective autophagy receptor SQSTM1 (sequestosome 1, SQSTM1)/p62 can mediate the degradation of kelch-like ECH-associated protein 1 (KEAP1), thereby releasing the transcription factor Nrf2. Promote the expression of antioxidant genes [85]. During this process, DAXX directly interacts with SQSTM1/p62 through residues 182–230. Expression of the N-terminal fragment (1–250 aa) is sufficient to promote p62 oligomerization and enhance its liquid–liquid phase separation (LLPS), resulting in the formation of larger SQSTM1 condensates. Functionally, this mechanism contributes to oxidative stress resistance: upon treatment with 300 μM H_2_O_2_, the percentage of PI-positive cells increased from 27.2% in wild-type cells to 48.4% in DAXX-knockout cells, indicating a substantial loss of cytoprotection in the absence of DAXX [86]. These condensates sequester KEAP1 in the cytoplasm, preventing KEAP1-mediated ubiquitination and degradation of Nrf2. This allows Nrf2 to stabilize and translocate into the nucleus, where it activates the transcription of downstream antioxidant genes [86]. Further studies have shown that this process does not depend on the PB1 domain of SQSTM1, but on the specific site of DAXX to enhance protein interaction [87,88]. Notably, during the regulation of reactive oxygen species (ROS) clearance, DAXX not only influences metabolic homeostasis but also exerts profound effects on the process of cellular senescence, suggesting a dual role that bridges metabolism and aging (Figure 3).

## 4. The Role of DAXX in the Aging Process

**Figure 4:**
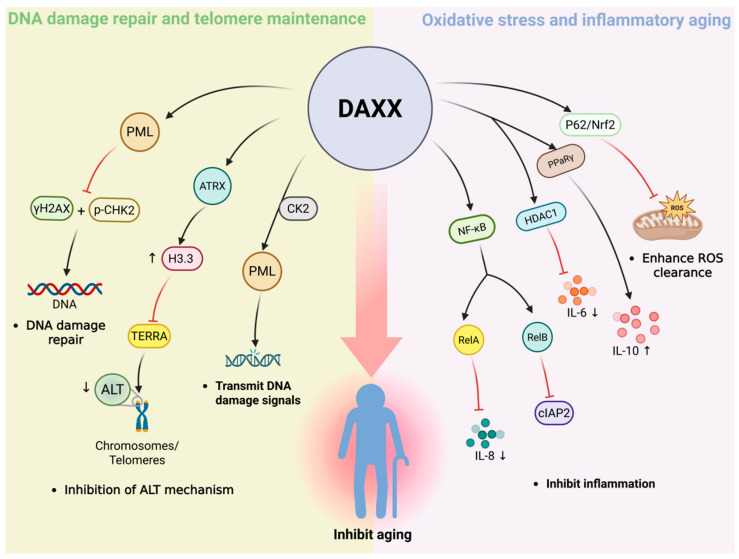
DAXX contributes to aging-related processes by regulating genome stability, telomere maintenance, oxidative stress, and inflammatory signaling. In the nucleus, DAXX associates with PML nuclear bodies and cooperates with ATRX to facilitate H3.3 deposition during DNA damage response and repair. At telomeres, the DAXX–ATRX complex restrains aberrant alternative lengthening of telomeres by limiting TERRA expression. Under oxidative stress, DAXX enhances antioxidant defense through activation of the p62–Nrf2 pathway. DAXX also suppresses aging-associated inflammatory signaling by modulating NF-κB activity and limiting IL-6 transcription. Black arrows indicate the direction of the proposed pathway/relationship. Red T-bars denote inhibitory effects. ↑ and ↓ indicate increased and decreased levels, respectively. Background colors separate functional modules and are for visualization only.

As age increases, the tissues and cells of the body gradually enter a state of aging. Aging is a process of functional degeneration over time, characterized by the accumulation of DNA damage, declining repair efficiency, and changes in chromatin structure [89]. Telomeres progressively shorten, making cells more susceptible to senescence [90]. At the same time, elevated levels of reactive oxygen species can damage proteins and lipids [91]. In addition, the long-term effects of chronic low-grade inflammation further disturb tissue homeostasis [92]. These mechanisms are intertwined, making aging highly complex and heterogeneous, and providing a fundamental perspective for revealing key regulatory factors and potential intervention strategies. At the same time, DAXX, as a chromatin and stress-related regulatory protein, is gradually being concerned about its potential role in the aging process (Figure 4).

### 4.1. DNA Damage Repair

DNA damage is an important factor in promoting cell aging [93]. As cells age, they become more prone to double-strand breaks, base modifications, and replication stress, which, if not repaired over time, disrupt genome stability and accelerate aging [89]. The DNA damage response includes recognition, signal transduction, and repair [89], but it is often impaired in senescent cells, leading to the accumulation of abnormal signals [94]. In recent years, in addition to classical repair factors, DAXX has been regarded as a potential key molecule linking DNA damage and aging because of its regulatory role in DNA damage response [7].

First is the damage sensing stage. When a double-strand break occurs, the histone variant γH2AX rapidly deposits at the damaged site, recruiting and activating checkpoint kinase 2 (CHK2) to amplify the initial signal [95]. DAXX plays a role in damage recognition by co-localizing with PML nuclear bodies in the nucleus, limiting the fragmentation signal [96]. PML nuclear body is an important DNA damage sensing structure in the nucleus [97]. Therefore, this localization indicates that the role of DAXX in this phase depends on its nuclear distribution. If DAXX is absent, the levels of γH2ax and p-CHK2 are significantly increased, and DNA damage signals are amplified, accompanied by the accumulation of tumor suppressor protein p53 and the overexpression of its downstream cyclin-dependent kinase inhibitor p21, which promotes cells to enter the senescence state in advance [96].

Next is the signal transduction stage. During this phase, PML nuclear bodies serve as key signaling platforms, cooperating with ATM/ATR to transmit DNA damage response signals [98]. Meanwhile, HDACs are recruited to induce transient chromatin compaction, facilitating the assembly of repair factors. DNA methyltransferase 1 (DNMT1) is rapidly and transiently recruited to DNA double-strand breaks, where it exerts a significant influence on the regulation of the DNA damage response and the rate of DSB repair [99]. In this step, DAXX binds to the PML nuclear body via its C-terminal SIM and is enriched in the nucleus by phosphorylation by the tyrosine kinase casein kinase 2 (CK2) [15]. This intranuclear enrichment is a prerequisite for its regulation of injury signaling. It has been shown that DAXX can form complexes with HDAC and DNMT1 in the nucleus [100]. It is suggested that it may assist the amplification of ATM/ATR signals by regulating the nuclear chromatin environment.

Finally, the stage of damage repair. At this stage, ATRX, a chromatin remodeling factor, is responsible for depositing H3.3 in heterochromatin and repetitive sequence regions to maintain the stability of nucleosome structure and provide the necessary platform for repair factors [101]. DAXX acts as a histone chaperone in the nucleus and cooperates with ATRX to efficiently deliver H3.3 to specific genomic regions, thereby ensuring the accuracy of repair [102]. This nuclear location-dependent histone loading process is a critical step in maintaining genome stability. If this mechanism is impaired, aberrant structural G-quadruplexes in DNA may gradually accumulate, leading to a decrease in repair accuracy and further amplification of the damage signal [103,104,105].

In conclusion, the localization of DAXX in the nucleus is the basis of its involvement in various aspects of DNA damage response. In the process of perception, transmission and repair, DAXX participates in signal recognition and transmission, and maintains chromatin homeostasis as a histone chaperone, relying on its distribution in nuclear structures such as nuclear bodies, chromatin and heterochromatin. Additionally, its interaction with regulatory factors such as HDACs and DNMT1 in the nucleus helps regulate the strength and timing of repair signals, playing a key role in maintaining genome stability and regulating cell senescence.

### 4.2. Maintenance of Telomeres

Telomeres, located at the ends of chromosomes, are composed of repetitive sequences and specific binding proteins, which can maintain the integrity of chromosomes [106]. With cell division, telomeres gradually shorten, and when they exceed the critical threshold, they will trigger DNA damage response and push cells into the aging state [90]. The stability of telomeres depends on factors such as telomere length, structural protection, and chromatin modification [90]. This also provides an entry point for DAXX to link telomere maintenance to the process of cell senescence.

In the process of telomere elongation, telomeric repeat-containing RNA (TERRA) can form telomeric R-loops, promote homologous recombination repair mechanisms that drive alternative telomere elongation (alternative lengthening of telomeres, ALT) [107]. Decreased levels of H3K9me3 at telomeres have been shown to significantly enhance ALT activity [108], suggesting a negative correlation between heterochromatin modifications and ALT [109,110]. DAXX forms a complex with ATRX at telomeric chromatin and suppresses inappropriate TERRA-associated recombination, thereby restraining aberrant ALT activity and preserving telomeric heterochromatin and genome stability [111]. This mechanism ensures telomere integrity without broadly impairing physiological telomere maintenance. The stability of telomere structure depends on TIN2 (TRF1-interacting nuclear factor 2) [112].When TIN2 is absent, the DAXX/ATRX complex accumulates abnormally at telomere ends and detaches from telomeres during cell differentiation [113], which in turn induces DNA damage and impedes differentiation. Because the DAXX/ATRX complex is capable of both depositing H3.3 and promoting H3K9me3 modification [19], its localization within telomeric chromatin is not only a prerequisite for the execution of these functions but also suggests that, in TIN2-deficient settings, the abnormal accumulation of DAXX/ATRX at telomeres is consistent with a compensatory increase in telomeric heterochromatin reinforcement; whether this compensation is functional requires further validation.

### 4.3. Oxidative Stress and Inflammatory Aging

Oxidative stress is considered to be an important driving force after DNA damage and telomere dysfunction during aging, and its core mechanism is that intracellular ROS levels continue to rise to break the homeostasis [93]. Excessive ROS can damage proteins, lipids and nucleic acids, and accelerate aging [91]. In this process, DAXX can activate p62–Nrf2 pathway through LLPS and enhance ROS scavenging capacity [86]. This mechanism echoes the mode of action in metabolic stress, suggesting that DAXX closely links metabolic stress to the driving force of aging by regulating the Nrf2–ROS network.

Under the background of continuous oxidative stress, the aging process is often accompanied by “inflammatory aging”, which is characterized by low but persistent chronic inflammation [114]. It is mainly driven by the long-term release of senescence-associated secretory phenotype (SASP) factors (such as IL-6, IL-8, IL-1β, etc.) [92], which can amplify damage signals between cells and accelerate functional degradation. In this process, sustained activation of the NF-κB (nuclear factor kappa-light-chain-enhancer of activated B cells) signaling pathway is considered a key molecular basis for maintaining chronic low-grade inflammation and amplifying aging-associated tissue damage [115]. Studies have shown that DAXX can regulate both the canonical and non-canonical NF-κB pathways: on the one hand, DAXX can bind to NF-κB p65 (RelA) and inhibit its acetylation level and DNA binding activity, thereby blocking the transcription of pro-inflammatory genes such as IL-8 and IκBα [116]; On the other hand, DAXX can also inhibit the expression of its downstream target gene cIAP2 by interacting with RelB, thus limiting the continuous activation and diffusion of inflammatory signals [117]. During aging, chronic inflammatory activation of microglia is considered to be the main manifestation of inflammatory aging in the central nervous system [118]. Against this background, studies on neuroinflammation-related models have further revealed its specific mode of action: in Pdcd4-deficient microglia, DAXX is more likely to localize in the nucleus and enhance its interaction with PPARγ, thus promoting the transcription of anti-inflammatory factor IL-10; Under LPS-stimulated conditions, this regulatory axis reduces inflammatory cell infiltration in neural tissue and alleviates neuroinflammation-related phenotypes [119]. In addition, at the SASP molecular level, DAXX can also be enriched in the IL-6 promoter region and recruit the deacetylase HDAC1 to reduce the level of local histone H3 acetylation, thereby selectively repressing IL-6 transcription via an epigenetic mechanism [120], which helps prevent sustained expression of inflammatory amplification factors during aging. Overall, DAXX suppresses aging-associated inflammatory programs by inhibiting NF-κB activity and restricting SASP-related gene expression.

## 5. The Role of DAXX in Inflammation and Immune Homeostasis

Immune homeostasis is crucial for the body to resist infections, eliminate abnormal cells, and maintain balance. It relies on the synergy between innate and adaptive immunity, as well as the precise regulation of immune tolerance and surveillance mechanisms [121,122]. Maintaining immune homeostasis depends not only on the development and functional status of immune cells but also on the precise control of transcriptional regulation and chromatin state, which govern the expression threshold of immune-related genes [123]. When immune regulation becomes imbalanced, abnormal activation or inhibition of immune responses can impair the body’s defense capabilities and contribute to the development of chronic diseases and tumors [121]. Recent studies have shown that DAXX, as a nuclear location-dependent chromatin regulatory protein, plays an important role in the multi-level regulation of immune homeostasis. By participating in interferon-associated immune responses [124,125], maintaining epigenetic silencing of endogenous retroviruses(ERV) [7,126], and regulating the activation threshold of key immune signaling pathways such as cGAS–STING [127,128], It affects the intensity, persistence and immune tolerance of innate and adaptive immune responses. These processes indicate that the maintenance of immune homeostasis is highly dependent on the cooperation of epigenetic and signal regulation in the nucleus, and DAXX is at the key intersection of this regulatory network.

Innate immunity, as the primary line of defense against exogenous pathogens and endogenous danger signals, relies on IFN-I signaling pathways to initiate defense responses rapidly in the early stage [129]. The PML nuclear body acts as a limiting factor in this process, inhibiting the transcriptional activation of the viral genome [130]. DAXX has been shown to be an important component of this mechanism by binding to the PML nuclear body and acting as a histone chaperone to deposit H3.3 onto the viral genome, prompting chromatization and heterochromatization, thereby inhibiting early viral gene transcription [131]. DAXX also exhibits extensive restrictions in RNA virus infections. For example, in the HIV-1 model, DAXX restricts viral reverse transcription and uncoating in a SUMO-dependent manner; notably, intact viral cores were reduced approximately three-fold in DAXX-depleted cells or cells expressing a DAXX SIM mutant compared with control cells [124]. In SARS-CoV-2 infection, DAXX has been identified as a potent host restriction factor. In the absence of IFNα, DAXX-mediated restriction resulted in a 42-fold reduction in viral RNA and a 62-fold reduction in infectious virus, highlighting its significant role in limiting viral replication [127]. A similar phenomenon was seen in a pancreas-specific DAXX knockout mouse model, in which the ERV pathway is significantly activated, and virus-like transcripts accumulate and stimulate innate immune receptors, leaving the body chronically overactivated [7].Thus, DAXX prevents the occurrence of immune hyperresponsiveness by maintaining ERV silencing and limiting viral replication.

Given that DAXX has been implicated in restricting multiple DNA and RNA viruses through distinct mechanisms, we compiled the key reported effects of DAXX on viral replication and the associated viral antagonism mechanisms in Table 1.

Adaptive immunity depends on the differentiation and functional maintenance of T and B cells, and its core feature is to provide lasting protection through antigen-specific recognition and immune memory [141]. T cell receptor (TCR) will trigger the activation and expansion of T cells after recognizing antigens, thus ensuring the strength of immune response [121]. It has been shown that T-cell specific deletion of DAXX results in a significant decrease in the number of peripheral mature T cells, and these cells are more susceptible to apoptosis after TCR activation, suggesting that DAXX plays a protective role in the survival of activated T cells and avoids premature attenuation of immune response [142]. Meanwhile, B cell differentiation determines the quality and persistence of antibody response, which is regulated by multiple transcription factors, including paired box protein 5 (Pax5), which is the core factor driving B cell lineage development [143]. Studies have shown that DAXX can assist Pax5 to perform both transcriptional activation and repression functions, thus playing an intermediate role in the early stage of B cell differentiation [144]. In addition, DAXX is also an essential factor in the IFN-α-mediated inhibitory pathway to protect pro-B cells from inhibition [145],thereby ensuring as to ensure the development process and functional maturity of B cells. These findings suggest that DAXX plays an important role in the maintenance of adaptive immune homeostasis by supporting the survival of T cells to maintain the persistence of immune responses and by participating in transcriptional regulation during the differentiation of B cells to promote their early development, thereby indirectly affecting antigen-specific recognition and the formation of immune memory.

For clarity and ease of reference, in vivo DAXX KO/cKO mouse models with clearly reported phenotypes are summarized in Table 2.

Immune tolerance is a key mechanism for the body to prevent the immune system from attacking its own components, once destroyed, it may lead to autoimmune diseases. Abnormally high expression of pro-inflammatory factors such as IL-6 can drive the immune system to attack autoantigens [147]. In view of the transcriptional repression of IL-6 expression by DAXX described above [120], the functional significance of this regulatory mechanism in the maintenance of immune tolerance has also been gradually concerned, suggesting that DAXX may be involved in regulating the tolerance threshold of immune response by limiting the continuous accumulation of pro-inflammatory signals. In addition, if hematopoietic stem cells are excessively biased towards granulocytic differentiation, it will lead to an increase in the proportion of pro-inflammatory cells [148] and destroy immune homeostasis; Given DAXX-mediated repression of IL-6 transcription [120,142], this mechanism links DAXX to the control of immune tolerance thresholds by limiting the persistent accumulation of pro-inflammatory signals. These findings suggest that DAXX participates in the maintenance of immune tolerance through multiple mechanisms and avoids the occurrence of autoimmune reactions.

Previous studies have demonstrated that DAXX exerts an inhibitory role in the regulation of inflammatory signaling [120]. Together with its function in immune cell survival [142], DAXX suppresses pro-inflammatory bias during hematopoietic differentiation and preserves immune-cell population diversity. These findings suggest that DAXX participates in the maintenance of immune tolerance through multiple mechanisms and avoids the occurrence of autoimmune reactions.

Tumor immunity depends on the effective recognition and elimination of tumor antigens by the immune system, and immune escape is a key mechanism for the long-term survival and expansion of tumor cells [149]. The cyclic GMP-AMP synthase (cGAS)–interferon gene stimulating factor (stimulator of interferon genes, STING) pathway plays a central role in tumor immunity. Its activation can induce the production of interferon and a variety of immune effector molecules, thereby promoting anti-tumor immunity [150]. In colorectal cancer, DAXX inhibits the cGAS–STING pathway by suppressing TBK1 and IRF3 phosphorylation, thereby weakening STING-dependent immune responses and reducing chemotherapy efficacy [151]. In the same study, irinotecan (CPT-11) and oxaliplatin (OXA) accelerated DAXX degradation and reduced nuclear DAXX speck formation; Daxx knockdown enhanced OXA-mediated tumor growth inhibition in CT-26 tumors in association with increased STING activation and immune responses, demonstrating that degradation of nuclear DAXX strengthens cGAS–STING signaling and improves chemotherapy response [151]. In the preceding sections, we discussed that DAXX promotes tumor progression in several cancer types, including breast and ovarian cancers through enhancement of lipid biosynthesis and small cell lung cancer through glycolytic reprogramming [5,18,68]. These findings suggest that DAXX supports tumor growth by facilitating metabolic adaptation in specific malignancies. However, these tumor-promoting effects are not universal; in gastric cancer, DAXX expression is reduced in advanced tumors, and restoring DAXX suppresses EMT and tumor growth [152]. Taken together, current evidence suggests that DAXX’s role in tumorigenesis is context-dependent and varies across different tumor types. Instead, its functional consequences seem to depend on the dominant biological context within each tumor, such as metabolic demands, chromatin regulatory state, and immune microenvironment. Differences in tumor subtype, experimental models, and the molecular status of DAXX may therefore contribute to the divergent observations reported in the literature. Similar principles of DAXX-mediated chromatin regulation are also evident in antiviral immunity. In viral infection studies, herpes simplex virus type 1 (HSV-1) uses its encoded immediate early protein ICP0 to degrade PML and MORC family protein 3, thereby disrupting the integrity of the PML-NB complex and relieving restrictions including DAXX, ultimately promoting transcriptional activation of viral genes [153]. Such disruption of PML-NB architecture suggests an important role for nuclear DAXX in maintaining immune-related chromatin states and gene accessibility.

**Figure 5:**
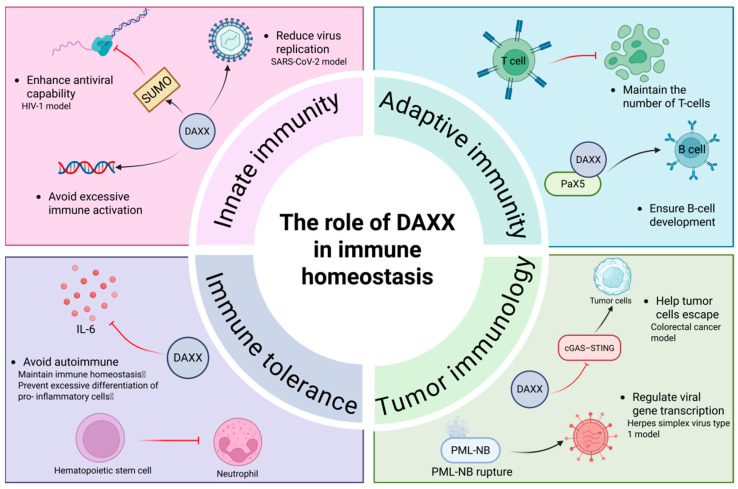
DAXX regulates immune homeostasis through chromatin-based and signaling-dependent mechanisms. In innate immunity, DAXX associates with PML nuclear bodies to promote chromatinization and silencing of viral genomes and endogenous retroviruses, thereby limiting excessive interferon responses. In adaptive immunity, DAXX supports T cell survival following TCR activation and cooperates with Pax5 during early B cell differentiation. DAXX also contributes to immune tolerance by repressing pro-inflammatory gene expression, including IL-6, and modulates tumor immunity by inhibiting activation of the cGAS–STING pathway. Black arrows indicate the direction of the proposed pathway/relationship. Red T-bars denote inhibitory effects. Colored panels are used to group functional modules for clarity and have no quantitative meaning.

Therefore, DAXX is involved in innate defense, lymphocyte homeostasis, immune tolerance and tumor surveillance. By regulating chromatin accessibility in the nucleus, participating in transcription complex assembly, and regulating signaling thresholds, DAXX establishes a dynamic balance between preventing over-activation of the immune response and maintaining an effective immune response. Its function is obviously context-dependent in different cell types and pathological environments, suggesting the complexity of this regulatory network. If future studies can further clarify the direct molecular links between DAXX and key immune pathways, it will help to reveal the relationship between DAXX and aging, infection and tumorigenesis, and may provide new ideas for the intervention of immune-related diseases (Figure 5).

Taken together, the evidence underscores the multifaceted and context-specific functions of DAXX. For clarity, the major DAXX-associated proteins described across metabolic, aging, and immune sections are summarized in Table 3.

## 6. DAXX-Associated Diseases and Potential Intervention Strategies

In recent years, DAXX has emerged as an important molecular factor implicated in a broad spectrum of pathological conditions. Aberrant DAXX function has been documented in metabolic disorders, aging-associated pathologies, and immune and inflammatory dysregulation. Increasing evidence indicates that, depending on the disease context, DAXX modulates disease progression through its effects on energy metabolism, fibrotic signaling cascades, and transcriptional control of inflammatory programs. A systematic synthesis of DAXX-related evidence across disease categories is therefore warranted to clarify the key molecular axes involved and to delineate potential therapeutic avenues.

### 6.1. Metabolic Diseases

The functional relevance of DAXX in metabolic disorders has been substantiated in models of diet-induced obesity and hepatic fibrosis.

In high-fat-diet-induced obesity (diet-induced obesity, DIO) mouse models, DAXX was identified as a binding partner of the AMPK-related kinase MPK38/MELK. Mechanistically, DAXX enhances both the stability and kinase activity of MPK38. Restoration of DAXX expression via adenoviral delivery in DIO mice resulted in activation of MPK38 and markedly improved glucose and lipid metabolic parameters, underscoring the functional importance of the DAXX–MPK38 axis in maintaining metabolic homeostasis [62]. These findings position MPK38 as a critical regulatory node through which DAXX exerts metabolic control in obesity-associated dysfunction.

In hepatic fibrosis, reduced Daxx expression has been observed in fibrotic liver tissues from both human patients and mouse models. In vitro studies using TGF-β stimulation demonstrated that Daxx interacts with Smad2 and suppresses its acetylation, thereby attenuating Smad2 transcriptional activity and inhibiting epithelial–mesenchymal transition (EMT)-associated gene expression programs. In a thioacetamide-induced liver fibrosis model, therapeutic elevation of Daxx levels alleviated fibrotic phenotypes [154]. These data collectively indicate that Daxx modulates fibrotic remodeling of metabolic liver tissue through the TGF-β–Smad2 signaling axis.

Taken together, experimental evidence supports a mechanistically defined role for DAXX in metabolic disease contexts, primarily mediated through the DAXX–MPK38 and DAXX–Smad2 pathways, thereby providing a conceptual framework for further translational investigation.

### 6.2. Aging-Related Diseases

Protein aggregation is a central pathological hallmark of neurodegenerative disorders such as Alzheimer’s disease [155]. Recent work has identified DAXX as a polyD/E protein enriched in aspartic acid and glutamic acid residues, conferring a distinctive chaperone-like function [155]. Through its polyD/E domain, DAXX directly binds misfolded substrate proteins, suppresses the formation of aberrant aggregates, and facilitates the disassembly and refolding of pre-existing aggregates. Notably, this activity is ATP-independent and relies on the highly negatively charged region of DAXX to modulate folding intermediates [155]. Validation across multiple aggregation-prone protein models has established DAXX as an integral component of the cellular proteostasis network. Given the central role of aberrant protein aggregation in neurodegeneration, DAXX-mediated anti-aggregation and refolding mechanisms provide a defined molecular basis for understanding age-associated neuronal decline. Strategies aimed at modulating polyD/E domain activity, enhancing DAXX-dependent proteostasis capacity, or stabilizing its expression in neural tissues represent rational avenues for therapeutic exploration.

Beyond proteostasis, telomere integrity and chromatin stability constitute fundamental determinants of aging [156]. As a core component of the ATRX–DAXX–H3.3 complex, DAXX mediates the deposition of histone variant H3.3 at telomeric regions and contributes to heterochromatin maintenance [6]. Telomere dysfunction and chromatin remodeling imbalance are strongly associated with age-related tissue degeneration. By sustaining telomeric chromatin architecture, DAXX promotes genomic stability and suppresses aberrant transcriptional activation. This positions DAXX at a critical interface between chromatin homeostasis and age-associated genomic instability. Accordingly, preservation of the structural and functional integrity of the ATRX–DAXX–H3.3 axis may provide a mechanistic basis for interventions targeting aging-related degenerative disorders.

Collectively, DAXX contributes to aging-associated disease mechanisms through coordinated regulation of proteostasis and telomeric chromatin maintenance, thereby establishing a mechanistic platform for translational evaluation in age-related pathologies.

### 6.3. Immune-Related Diseases

Autoimmune polyendocrinopathy-candidiasis-ectodermal dystrophy (APECED) is a rare autoimmune syndrome caused by functional deficiency of the AIRE gene and is characterized by autoimmune destruction of multiple endocrine organs [157]. AIRE serves as a central transcriptional regulator of thymic immune tolerance, promoting the ectopic expression of peripheral tissue–specific antigens in medullary thymic epithelial cells to facilitate negative selection of autoreactive T cells [158]. DAXX has been shown to directly interact with AIRE and suppress its transcriptional activity [159]. Through modulation of AIRE-dependent gene expression programs, DAXX becomes integrated into the molecular circuitry governing central immune tolerance. The DAXX–AIRE interaction therefore represents a mechanistic node relevant to immune tolerance dysregulation and autoimmune pathogenesis.

In the context of viral infection, host intrinsic defense mechanisms restrict viral gene expression through promyelocytic leukemia (PML) nuclear body–associated proteins [160]. DAXX is a key structural and functional component of PML nuclear bodies and represses viral immediate-early gene transcription during the early phase of infection [132]. In human cytomegalovirus (HCMV) infection, the viral tegument protein pp71 induces proteasome-dependent degradation of DAXX, thereby relieving transcriptional silencing and facilitating immediate-early gene expression and viral replication [132]. Similarly, in adenovirus infection models, the early viral protein E1B-55K binds to DAXX and promotes its proteasomal degradation, leading to derepression of viral gene expression [138]. Moreover, during human adenovirus type 5 (HAdV5) infection, the DAXX/ATRX complex associates with viral genomes to restrict transcriptional activity; viral infection results in displacement of this complex, enabling activation of the viral transcriptional program [161]. These findings, validated across CMV and adenovirus systems, establish DAXX as a critical mediator of intrinsic antiviral defense and highlight viral targeting of DAXX as a conserved immune evasion strategy. Therapeutic approaches aimed at stabilizing DAXX within PML nuclear bodies, preventing virus-induced degradation, or preserving its association with viral genomes may further clarify its role in virus-associated disease contexts.

## 7. Conclusions

This review summarizes the biological characteristics of DAXX, focusing on its structure and function, and highlights the central role of its nuclear localization in coordinating various biological processes. As a nexus of chromatin regulation and signal integration, DAXX reshapes the transcriptional landscape, regulates epigenetic modifications, and modulates key signaling thresholds. This results in the formation of a continuous, highly integrated regulatory network across multiple systems, including energy metabolism, cellular aging, and immune balance. DAXX’s function extends beyond the regulation of a single molecular pathway; it provides both structural and functional support for maintaining cellular homeostasis and adapting to environmental changes through the coordination of spatial localization, chromatin state, and signal integration.

The versatility of DAXX is evident in its high degree of ‘environmental dependence’ and ‘bidirectionality’. In metabolic regulation, DAXX promotes lipid synthesis by activating SREBP1/2 and enhances fatty acid oxidation via MPK38, depending on the cell’s metabolic state. During aging, DAXX collaborates with ATRX in the nucleus to maintain telomere and genome integrity by stabilizing the deposition of H3.3, a key mechanism for delaying aging. Additionally, DAXX boosts cellular antioxidant capacity by activating the Nrf2 pathway, helping to counteract oxidative stress caused by aging and chronic inflammation. At the level of immune regulation, DAXX modulates the intensity and persistence of immune responses by fine-tuning the transcriptional programs of immune-related genes, and plays a role in the body’s defense against pathogen stimulation. More specifically, DAXX links metabolism, aging, and immunity through a shared stress-coupling cascade. DAXX-driven metabolic remodeling (e.g., through SREBP1/2- and MPK38-linked pathways) reshapes the cellular redox state. Under metabolic stress, DAXX activates the p62–Nrf2 axis to enhance ROS clearance, effectively buffering the oxidative stress that accelerates aging. Simultaneously, DAXX preserves telomere and genome integrity through the ATRX–DAXX–H3.3 pathway, limiting entry into senescence and the inflammatory output of the SASP (particularly IL-6), which directly influences immune response intensity, persistence, and tolerance thresholds. Finally, when genome instability and damage-derived DNA signals engage innate immune sensing, DAXX further modulates this interface by regulating cGAS–STING signaling, reinforcing its role as a molecular bridge from metabolic/aging stress cues to immune activation. It is this integrated function across multiple biological levels, such as metabolic homeostasis, aging regulation and immune homeostasis, that makes DAXX a key molecule in understanding the interweaving of these processes.

**Figure 6:**
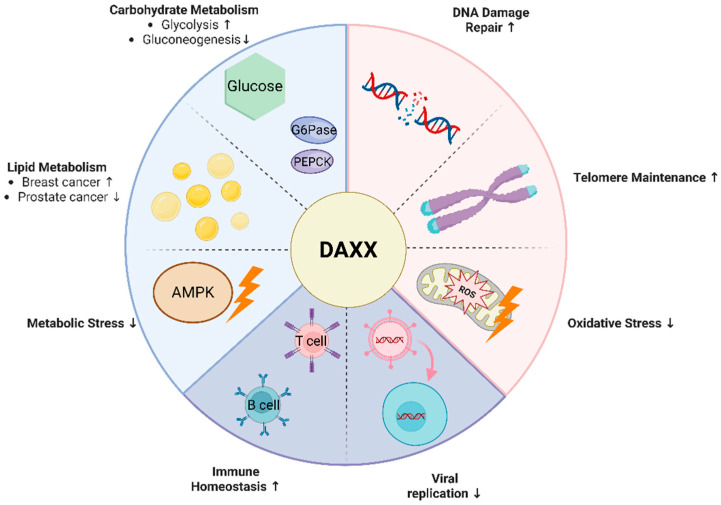
Schematic overview illustrating DAXX as an integrative regulator linking metabolic control, aging-associated stress responses, and immune homeostasis. Through context-dependent nuclear localization and protein interactions, DAXX coordinates transcriptional and signaling pathways involved in energy metabolism, genome stability, oxidative stress regulation, and immune signaling. ↑ and ↓ indicate increased and decreased activity/levels, respectively. Colors are used to group related functional modules in the schematic and have no quantitative meaning.

Therefore, further exploration of DAXX function will not only enhance our understanding of its role in both normal physiological and pathological conditions but also provide new insights into identifying common molecular roots of chronic diseases, such as obesity, diabetes, and neurodegenerative disorders. Future studies should focus on the specific molecular mechanisms of DAXX and core signaling pathways, such as NF-κB, and investigate how its functions differ across tissues and pathological environments. For example, determining whether selective inhibition of DAXX could restore cGAS–STING signaling and enhance anti-tumor immunity in colon cancer, or whether targeting DAXX-driven metabolic programs might attenuate tumor growth in metabolically dependent cancers, such as breast or ovarian carcinoma. By clarifying its role in aging and chronic diseases, this research will help transition DAXX from molecular mechanism studies to clinical applications, providing a theoretical foundation for precise interventions (Figure 6).

## Figures and Tables

**Table 1:** DAXX regulation of viral replication across different viruses.

Virus	Effect of DAXX on Viral Replication	Reported Step or Mechanism	Viral Countermeasure	Refs
HCMV	DAXX restricts viral immediate early gene expression and lytic infection	DAXX mediates intrinsic silencing of incoming viral genomes at nuclear bodies	pp71 promotes proteasome dependent loss of DAXX to relieve repression	[132,133]
HSV-1	DAXX restricts viral gene expression and replication, strongest when ICP0 is absent	ND10 proteins including DAXX repress viral transcription at early stages	ICP0 disrupts ND10 based restriction and counteracts repressive factors	[134,135,136]
EBV	DAXX restricts early infection unless antagonized	DAXX ATRX chromatin repression on incoming viral genomes at nuclear bodies	BNRF1 binds DAXX and disrupts the DAXX ATRX restriction complex	[136,137]
HAdV-5	DAXX restricts adenovirus replication and gene expression	DAXX imposes restrictions on viral growth and transcription	E1B 55K binds DAXX and induces proteasome dependent degradation of DAXX	[138]
VZV	DAXX restricts very early stages of replication	ND10 components including DAXX associate with viral genomes and limit early replication events	Viral antagonism of ND10 response is reported but a specific DAXX antagonist is not defined	[139]
HIV-1	DAXX restricts early infection	DAXX inhibits uncoating and reverse transcription through SUMO dependent interactions	A specific viral antagonist of DAXX is not defined in that study	[124]
SARS-CoV-2	DAXX restricts viral replication and infectious virus production	DAXX targets an early post entry step and relocalizes to cytoplasmic replication sites	PLpro induces proteasome dependent degradation of DAXX	[127]
HPV-18	DAXX supports viral DNA replication and early transcription in the tested system	DAXX contributes to replication focus function in cell-based replication assays	A specific viral antagonist of DAXX is not defined in that study	[140]

**Table 2:** Representative in vivo DAXX loss-of-function mouse models (KO/cKO) and key phenotypes.

Model (Mouse)	Type	Key In Vivo Phenotype (s)	Refs
Daxx germline knockout	Global knockout	Early embryonic lethality with extensive apoptosis	[146]
T cell specific DAXX conditional knockout	Conditional knockout in T cells	Reduced peripheral mature T cell numbers. Increased apoptosis after T cell receptor activation. Fas induced apoptosis remains intact	[142]
Pancreas lineage DAXX conditional knockout in vivo	Conditional knockout in pancreas lineage	Maintains endogenous retroviral silencing. Restricts cellular plasticity. Impaired pancreas recovery under inflammatory stress	[7]

**Table 3:** DAXX-associated proteins and regulatory mechanisms in metabolism, aging, and immunity.

Partner	Evidence	Context	Outcome	PTM	Refs
SREBP1/2	Physical interaction	Lipid metabolism	Up-regulates HMGCR/HMGCS1 and promotes cholesterol accumulation; promotes fatty acid synthesis.		[5,42,43,44]
AR	Physical interaction	Lipid metabolism	Inhibits SREBP2 activation and reduces downstream synthetic genes.	SUMOylation	[28,49,50]
Caveolin-1	Functional association	Lipid metabolism	Promotes cholesterol efflux and prevents lipid deposition.	—	[57,58,59]
MPK38	Physical interaction	Lipid metabolism	Increases fatty acid oxidation; reduces hepatocyte lipid droplets; reduces serum triglyceride and total cholesterol.	—	[62]
GLUT4 complex	Physical interaction	Glucose metabolism	Regulates GLUT4 membrane translocation and glucose intake; DAXX loss retains GLUT4 in endosomes and impairs glucose transport.	SUMOylation	[68,69,70]
PEPCK, G6Pase	Functional association	Glucose metabolism	Restoring the expression of DAXX can inhibit the expression of PEPCK and G6Pase, reduce the level of liver gluconeogenesis and improve the level of blood glucose [61].	—	[62]
ASK1	Functional association	Antioxidant Response	Promotes apoptosis to remove severely damaged cells.	—	[9]
SQSTM1/p62 complex	Physical interaction	Antioxidant Response	Stabilizes Nrf2 and promotes nuclear translocation to activate antioxidant genes.	—	[85,86]
PML-NB complex	Physical interaction	DNA damage repair	DAXX absence increases γH2ax/p-CHK2, p53 and p21, promoting premature senescence.	—	[96,97]
		DNA damage repair	Intranuclear enrichment supports regulation of injury signaling.	Phosphorylation by CK2	[15]
		Innate immunity	Inhibits early viral gene transcription.	—	[131]
ATRX	Physical interaction	DNA damage repair	Ensures repair accuracy and genome stability.	—	[111]
		Maintenance of telomeres	Limits aberrant ALT activation.	—	[111]
NF-κB (RelA)	Physical interaction	Inflammatory aging	Blocks transcription of pro-inflammatory genes.	Acetylation	[116]
NF-κB(RelB)	Physical interaction	Inflammatory aging	Inhibits cIAP2 expression and limits inflammatory signal activation/diffusion.	—	[117]
PPARγ	Physical interaction	Inflammation aging	Promotes IL-10 transcription; reduces inflammatory infiltration and alleviates phenotypes under LPS stimulation.	—	[119]
Pax5	Functional association	Adaptive immunity	Intermediate role in early B-cell differentiation.	—	[144]
cGAS–STING	Functional association	Tumor immunity	Impairs immune effector molecule production and promotes tumor immune escape.	Phosphorylation of TBK1 and IRF3	[151]

## Data Availability

No new data were created or analyzed in this study.

## Abbreviations

The following abbreviations are used in this manuscript:

4HB	Four-helix bundle
ACC	Acetyl-CoA carboxylase
ALT	Alternative lengthening of telomeres
AMPK	AMP-activated protein kinase
AR	Androgen receptor
ASK1	Apoptosis signal-regulating kinase 1
ATR	Ataxia telangiectasia and Rad3-related protein
ATRX	Alpha-thalassemia/mental retardation syndrome X-linked
AREs	Androgen response elements
CALML3-AS1	CALML3 antisense RNA 1
cGAS	Cyclic GMP-AMP synthase
CHK2	Checkpoint kinase 2
CK2	Casein kinase 2
CPT1A	Carnitine palmitoyltransferase 1A
DAXX	Death domain-associated protein 6
DGAT	Diacylglycerol acyltransferase
DNMT1	DNA methyltransferase 1
DSB	DNA double-strand break
DHT	Dihydrotestosterone (DHT
ERV	Endogenous retrovirus
FASN	Fatty acid synthase
G6Pase	Glucose-6-phosphatase
GLUT	Glucose transporter
H3.3	Histone H3 variant 3
H3K4me3	Trimethylation of histone H3 lysine 4
H3K9me3	Trimethylation of histone H3 lysine 9
HDAC	Histone deacetylase
HBD	Histone-binding domain
HK	Hexokinase
HMGCR	3-hydroxy-3-methylglutaryl-CoA reductase
HMGCS1	3-hydroxy-3-methylglutaryl-CoA synthase 1
IFN-I	Type I interferon
JNK	c-Jun N-terminal kinase
KEAP1	Kelch-like ECH-associated protein 1
KIF5B	Kinesin family member 5B
LDLR	Low-density lipoprotein receptor
LLPS	Liquid–liquid phase separation
MASLD	Metabolic dysfunction-associated steatotic liver disease
MASH	Metabolic dysfunction-associated steatohepatitis
MDM2	Mouse double minute 2 homolog
MPK38	Maternal embryonic leucine zipper kinase (MELK)
mTOR	Mechanistic target of rapamycin
NF-κB	Nuclear factor kappa-light-chain-enhancer of activated B cells
Nrf2	Nuclear factor erythroid 2-related factor 2
PB1	Phox and Bem1 domain
PEPCK	Phosphoenolpyruvate carboxykinase
PFK1	Phosphofructokinase 1
PK	Pyruvate kinase
PML	Promyelocytic leukemia protein
PML-NB	Promyelocytic leukemia nuclear body
PPARγ	Peroxisome proliferator-activated receptor gamma
RASSF1C	Ras association domain family member 1C
ROS	Reactive oxygen species
SCAP	SREBP cleavage-activating protein
SCD1	Stearoyl-CoA desaturase 1
S1p	Site 1 protease
S2p	Site 2 protease
SIM	SUMO-interacting motif
SQSTM1	Sequestosome 1
SREBP	Sterol regulatory element-binding protein
STING	Stimulator of interferon genes
SUMO	Small ubiquitin-like modifier
TAG	Triacylglycerol
TERRA	Telomeric repeat-containing RNA
TIN2	TRF1-interacting nuclear factor 2
TCR	T cell receptor
